# Immediate 3D Skull Changes Following 3D-Guided Midpalatal Piezocorticotomy-Assisted MARPE: Case Report

**DOI:** 10.3390/dj14010024

**Published:** 2026-01-04

**Authors:** Svitlana Koval, Viktoriia Kolesnyk, Daria Chepanova

**Affiliations:** 1 DrKoval Orthodontics, Boca Raton, FL 33431, USA; info@drssk.com; 2 Department of Obstetrics, Gynecology and Reproductive Sciences, Yale School of Medicine, Yale University, New Haven, CT 06510, USA; viktoriia.kolesnyk@yale.edu

**Keywords:** MARPE, guided midpalatal piezocorticotomy, adults, 3D analysis, cephalometrics, upper airway

## Abstract

**Background/Objectives**: Mini-Screw-Assisted Rapid Skeletal Expansion (MARPE) appliances have been widely used for maxillary skeletal expansion in non-growing subjects and adolescents with a fused midpalatal suture. The current case report describes the immediate 3D cephalometric changes in the skeletal and soft tissue parameters, along with upper airway volume, shape, and dimensions, in a patient with Skeletal Class I anterior underbite. **Methods:** The pre- and post-expansion full-face Cone-Beam Computed Tomograms (CBCTs) of a 19-year-old patient who underwent 3D-guided midpalatal piezocorticotomy-assisted MARPE were compared and analyzed using 3D cephalometric software. Both CBCT volumes were re-oriented relative to the Frankfurt horizontal plane (FHP) to accommodate postural changes. **Results:** The total upper airway volume and minimum upper airway cross-section increased after expansion. The nasal base plane (ANS–PNS) rotated in all three spatial planes, including the sagittal plane (anterior downward and posterior upward rotation, with the center of rotation around the maxillary center of rotation) and the vertical plane (upward rotation on the left side). The maxillary canine and molar cant planes rotated around the center of rotation in the midface, with left upward and right downward rotation. The orientation of the ANS–PNS plane changed due to the leftward rotation of the ANS, with the center of rotation approaching the PNS. Cervical curvature improved from kyphotic to lordotic immediately following expansion. **Conclusions:** Three-dimensionally guided midpalatal piezocorticotomy-assisted MARPE has been shown to produce midfacial changes in all three spatial planes when evaluated via 3D cephalometric analysis. Comprehensive observational studies are necessary to analyze these changes and their effects for different skeletal classifications.

## 1. Introduction

Mini-Screw-Assisted Rapid Skeletal Expansion (MARPE) appliances have been widely used for maxillary skeletal expansion in non-growing subjects and adolescents with a fused midpalatal suture [1,2,3]. Auxiliary techniques have been suggested to augment the midpalatal suture separation procedure in adult patients [4,5].

Nasal cavity measurements for MARPE are typically performed in the area around the ANS, PNS, nasal septum deviation angle [6], and pharyngeal volumes. Nasal respiratory parameters were systematically reviewed by Arqub and coauthors [7], who did not find any correlation between nasal airflow and the transverse anatomical measurements of the upper airway after MARPE. Cantarella and colleagues showed a statistically significant increase in nasal cavity volume and nasopharyngeal dimensions following MARPE [8].

Most authors agree regarding the correlation between the upper airway shape and skeletal classification [9,10]. For example, Skeletal Class II patients show a lower position of the hyoid bone, with a smaller minimum oropharyngeal airway cross-section; meanwhile, those categorized under Skeletal Class III exhibit smaller nasopharyngeal airway dimensions and a higher position of the hyoid bone.

One study attributed forward head posture to compensation for decreased upper airway dimensions in the context of the prevalence of anterior Temporomandibular joint (TMJ) disk displacement [11]. The authors of the study referred to Skeletal Class II as a prerequisite for anterior cervical extension as part of an effort to achieve upper airway size compensation.

Paredes and colleagues [12] suggested novel angular measurements to assess the forward–outward rotation of the maxillary complex with Maxillary Skeletal Expander (MSE) treatment in the coronal plane. A study by Cantarella [13] and colleagues supported the idea of clockwise mandibular rotation with MSE treatment.

The primary aim of the current case study is to analyze the 3D cephalometric and airway changes in a patient with a Skeletal Class III tendency, underdeveloped midface, and anterior crossbite who underwent initial treatment with 3D-guided midpalatal piezocorticotomy-assisted MARPE. The secondary aim of this study is to evaluate the effect of the 3D-guided midpalatal piezocorticotomy-assisted MARPE procedure on the position of the palatal plane, as defined by the ANS-PNS plane, through 3D and 2D cephalometric measurements.

## 2. Materials and Methods

A 19-year-old male patient with the chief complaint of an underdeveloped midface and underbite presented for treatment with 3D-guided midpalatal piezocorticotomy-assisted custom MARPE [5]. Further orthodontic treatment with a direct printed aligner is to be provided after the initial expansion and will not be discussed in this case report.

**Figure 1:**
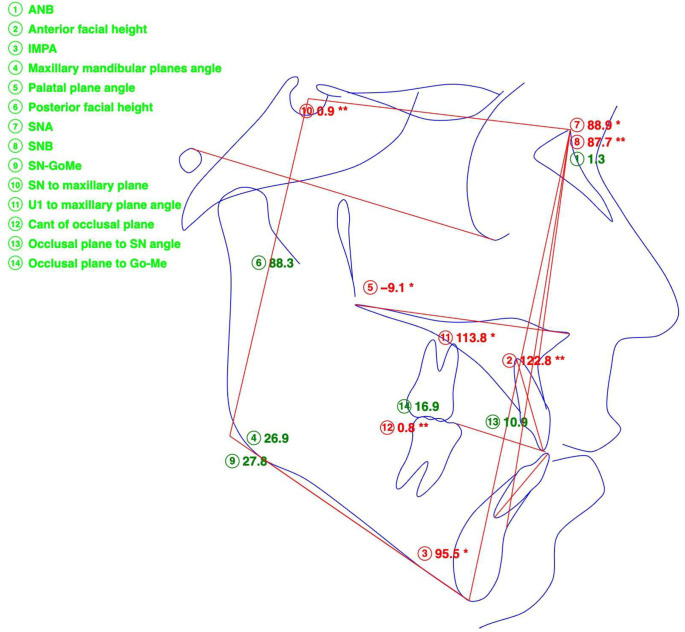
Lateral cephalometric tracing before treatment, indicating Skeletal Class I according to the ANB angle. Brachyfacial facial pattern with increased buccal inclination of maxillary and mandibular incisors and anterior crossbite. Numeric values marked with * lie within one standard deviation (SD) from the normal values, ** lie within two SD. Number identified in red deviate from the normal values by one or two SD.

Initial records included a CBCT of the head and neck. A full-face CBCT was obtained using a Planmeca Viso G7 (Planmeca, Helsinki, Finland) at 30 Sv^−6^ and 100 kV, with a 600-Voxel ultra-low-dose (ULD)-setting slice of a 19″ × 19″ sensor area, while the patient sat in an upright position with natural head posture and occipital head support. A pre-treatment lateral cephalometric radiograph was rendered from the CBCT, traced, and evaluated (Table 1, Figure 1).

Consent for this study was obtained before treatment.

The three-dimensional guide for the midpalatal piezocorticotomy was three-dimensionally designed and printed based on the patient’s initial CBCT and intraoral scan imaging. The precise positions of the incision notches and the depth of incision were calculated.

**Figure 2:**
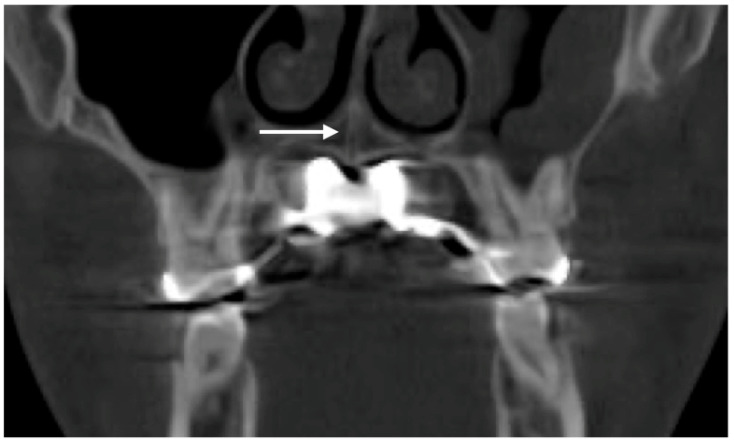
A coronal view of the nasal base floor at the level of the maxillary first molars depicting the location and depth of the piezocorticotomy incision and its relationship to the nasal septum’s position (white arrow). The 3d-guided piezocorticotomy is conducted with the aim of determining the location of the incision directly under the septum to ensure symmetrical disarticulation of the midpalatal suture.

The patient underwent the 3D-guided midpalatal piezocorticotomy procedure and custom MARPE positioning under topical anesthesia, namely 20% Benzocaine topical anesthesia and 0.5% 1:200 k epi Marcaine infiltration anesthesia. The piezocorticotomy cuts were made immediately after anesthesia onset with a Mectron piezotome (Mectron, Hilliard, OH, USA) and individually designed based on the 3D-printed piezocorticotomy guide. An ULD CBCT of the maxillary area was taken to confirm the depth and location of the piezocorticotomy incision along with the custom MARPE screw positioning (Figure 2).

While custom MARPE is a widely adopted modality for the treatment of bilateral posterior crossbite, unilateral crossbite, and a skeletally narrow maxilla, recent clinical observations support the use of this method for treating maxillary retrusion and Skeletal Class III characteristics in fully grown individuals. The novel 3D-guided piezocorticotomy-assisted MARPE approach was shown to predictably support greater midpalatal separation, reducing the risk of perimaxillary fractures [5]. Forward movement of the midface with up to 5.5 mm of midpalatal suture separation was reported by the same authors [14].

**Figure 3:**
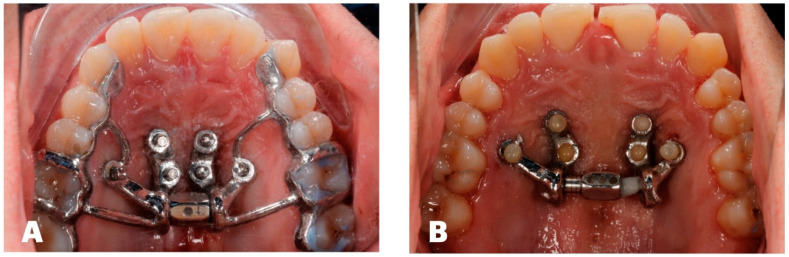
Pre-expansion view of the custom MARPE appliance (**A**) and post-expansion view with the power screw and framework arms removed, with additional composite bonding on the mesial surfaces of teeth #8 and 9 for esthetic purposes (**B**).

The custom MARPE design included a 12 mm power screw (TigerDental, Horbranz, Austria) with 2-turn/day activation until the initial split (14 days), 1-turn/day activation for the subsequent 3 weeks (days 15–36), and 1-turn/3-day activation for another 3 weeks (days 37–58). The expansion stage was initiated immediately at the beginning of treatment and lasted 58 days in total. Once desired facial changes were achieved, the expansion phase was deemed complete and the arms of the custom MARPE device were removed, with all records taken (Figure 3).

**Figure 4:**
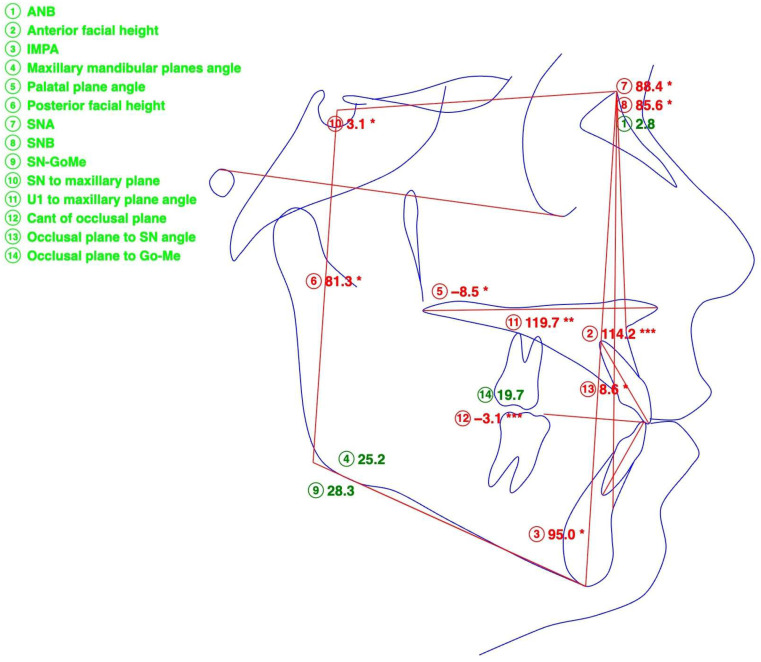
Lateral cephalometric tracing after expansion, indicating Skeletal Class II tendency according to the ANB angle. Anterior crossbite is resolved, maxillary incisor inclination is increased, and counter-clockwise palatal-plane rotation (FH-ANS-PNS) is evident. Numeric values marked with * lie within one standard deviation (SD) from the normal values, ** lie within two SD. Number identified in red deviate from the normal values by one or two SD.

Post-expansion records included a CBCT taken with identical parameters to those initially employed (19″ × 19″ sensor area, Planmeca Viso G7, NHP, Finland, Helsinki). A post-expansion lateral cephalometric radiograph was rendered for the comparison with the initial records (Figure 4, Table 2).

Pre- and post-expansion CBCTs were processed using NemoFab software (Nemotec, Madrid, Spain, version 2025), re-orienting the head’s position to the Frankfurt horizontal plane, evaluated relative to the true horizontal and true vertical planes [15,16], and compared.

## 3. Results

Both CBCT scans were taken in the low-dose setting (400 μm) and digitally analyzed using NemoFab software in all spatial planes to detect any changes attributable to 3D-guided midpalatal piezocorticotomy-assisted MARPE. Software calibration was performed prior to the study. The primary investigator (SK) performed repeated measurements of both CBCTs and lateral cephalometric radiographs at an interval of two weeks.

**Figure 5:**
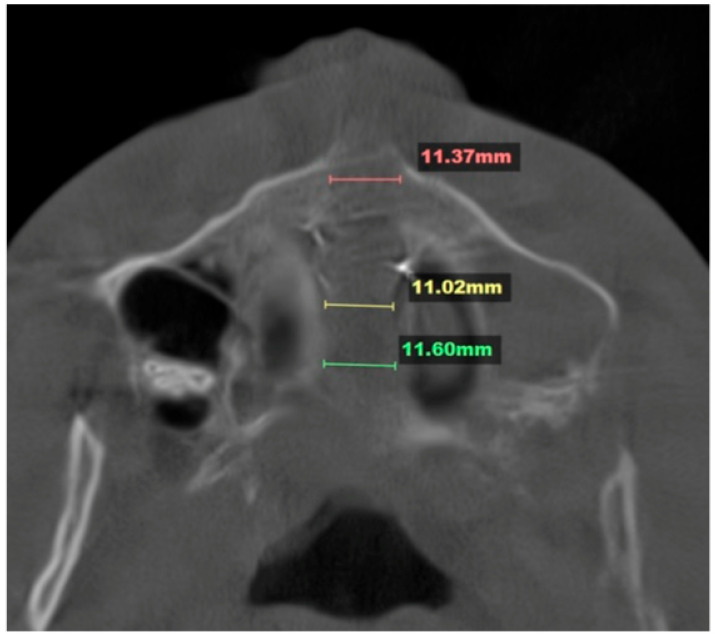
Disarticulation of the midpalatal suture with parallel separation at the level of the ANS and PNS.

The midpalatal disarticulation resulted in parallel separation of the midpalatal suture at all points, including the ANS and PNS (Figure 5).

The 3D cephalometric measurements were grouped as follows:

i. Sagittal: ANS to TVL; Mx incisor R to TVL; Mx incisor L to TVL; upper-right canine to TVL; upper-left canine to TVL; upper-right molar to TVL; upper-left molar to TVL; pogonion to TVL; B-point to TVL; PNS to TVL (Table 1).
ii. Canting: Mx33 cant.
iii. Width difference: width difference, Mx molar; width difference, Zyg arch; width difference, Lat Orb rim.
iv. Transverse: Mx molar R width; Mx molar L width; Zyg arch R width; Zyg arch L width; Lat Orb rim R width; Lat Orb rim L width.
v. Vertical: Mx incisor height; Mx canine R height; Mx canine L height; Mx molar R height; Mx molar L height; pogonion height; PNS height, menton height.
vi. Soft tissue: RChkbone; LChkbone; Rnasalbase; Lnasalabase.
vii. Airway measurements (Table 3): upper airway total volume; upper airway min cross-section (as described by Echarri and coauthors [17]).

The midpalatal suture disarticulation was measured at three points: the ANS, the middle third of the suture, and the PNA. The mean disarticulation was 11.33 mm (Figure 5).

The direction and extent of landmark translation in three spatial planes are outlined in Table 4. The overall vector of landmark displacement in the sagittal plane was oriented toward the TVL (forward), decreasing the distance between the landmark and the TVL (depicted as negative values).

The canting value remained negative but decreased by 1.1 degrees (indicating that the left side was lower than the right side), as seen through 3D evaluation.

Positive width-difference values denote a predominance of the right side, while negative values denote a predominance of the left side.

On the left side, an increase in the molar width and the zygomatic arch from the midfacial midline was observed, while on the right side, an increase in the lateral orbital rim width was noted.

The height values showed a similar vector oriented toward downward translation through an increase in the distance from the THP and the points on the surface of the anterior and posterior teeth. A right-side downward translation occurred, while on the left side, both canine and molar translation occurred in the opposite direction. Mandibular landmarks followed a similar pattern of downward translation, but to a lesser extent.

ANS–PNS-plane rotation occurred in a downward direction in the area around the ANS, occurring in an equal and opposite direction in the area around the PNS. While the PNS underwent upward translation, the occlusal surface of only the left first molar and canine followed this pattern. This is in agreement with the increase in the SN-ANS-PNS angle from 0.9 to 3.1 degrees on the lateral cephalometric tracings.

Soft tissue landmarks underwent significant changes with the improvement in lower lip prominence following backward mandibular rotation.

In terms of upper airway changes, both the total volume and minimum cross-section increased (Table 5).

**Figure 6:**
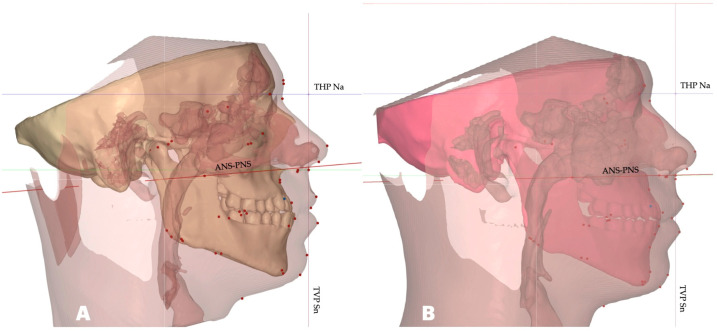
Inclination of the ANS–PNS plane before (**A**) and after (**B**) midfacial expansion, with both volumes oriented relative to the FHP. Inclination of the ANS–PNS is measured relative to the THP. The orientation of the ANS-PNS plane has changed after the procedure resulting in rotation of the ANS-PNS plane in sagittal plane. Both volumes are oriented relative to the THP-Na and TVP Sn planes.

As shown in Figure 6, the position of the ANS–PNS plane is sagittal.

**Figure 7:**
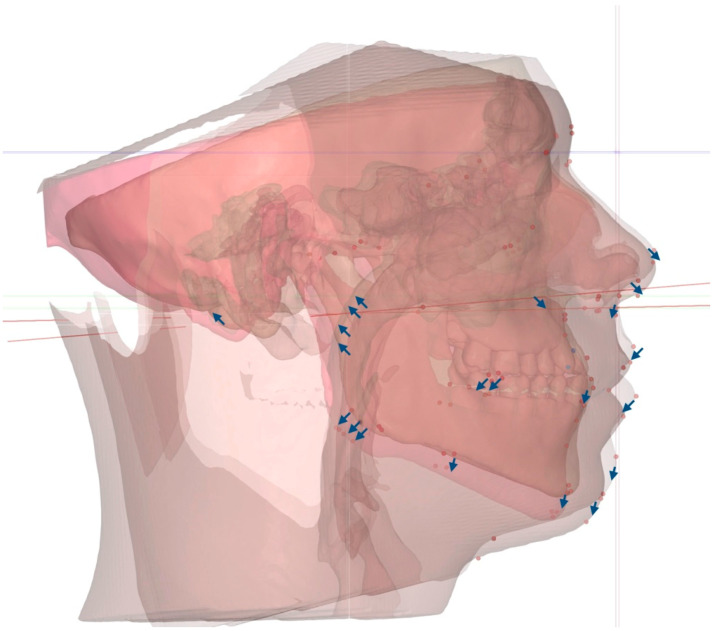
Total vectors of the 3D movements of the facial skeletal structures with 3D-guided midpalatal piezocorticotomy-assisted MARPE. The rotation vectors are marked with blue arrows.

The total vectors of the 3D changes introduced via 3D-guided midpalatal piezocorticotomy-assisted MARPE are represented in Figure 7.

**Figure 8:**
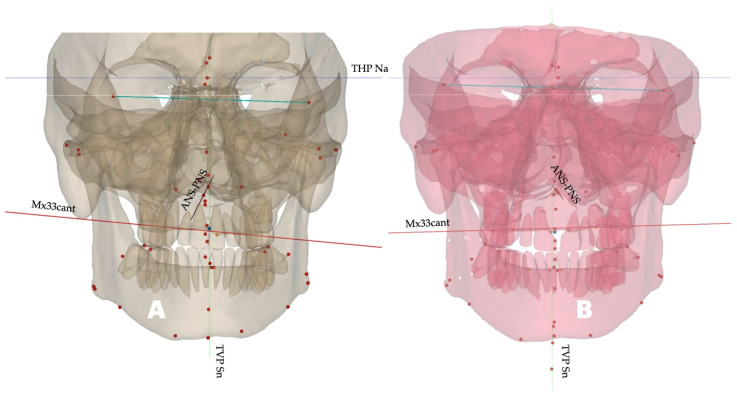
Pre- (**A**) and post-expansion (**B**) orientation of the ANS–PNS plane and maxillary cant plane, showing the distinct rotation of both. The orientation of the ANS–PNS plane revealed the left-side rotation of the plane, with the center of rotation at the PNS, whereas the maxillary canine plane rotated left-side up, with the center of rotation at the midface.

**Figure 9:**
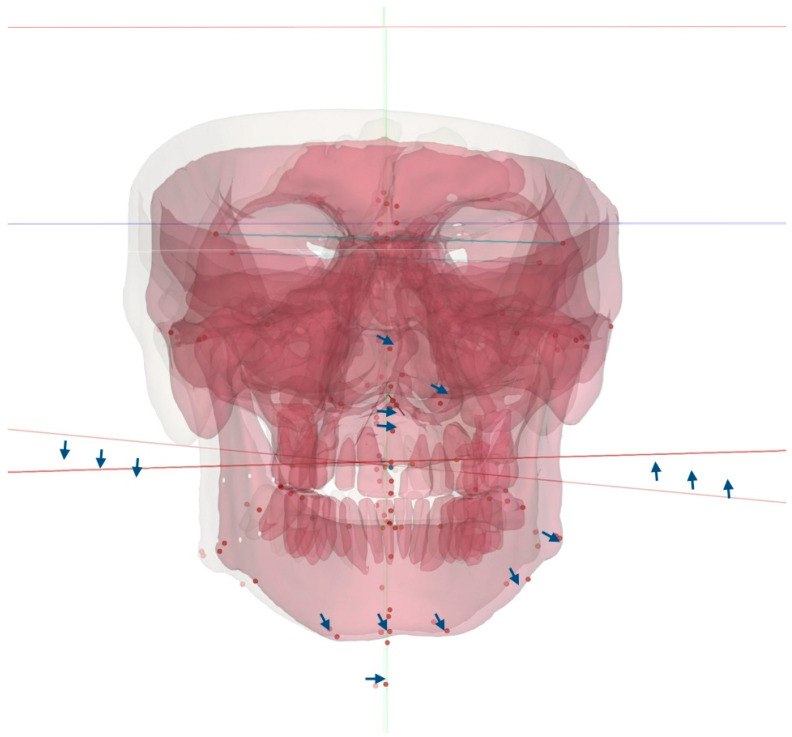
Total vectors of rotation in the axial plane, with superimposition on the true horizontal plane (THP at Na) and midfacial plane, showing the migration of the nasal septum and expansion toward the left lateral nasal wall, the rotation of the maxillary canine plane around the center of rotation at the midface, and the rotation of the ANS–PNS, favoring the left side, with the center of rotation at the PNS. The rotation vectors are marked with blue arrows.

Post-expansion changes and total vectors in the axial plane can be seen in Figure 8 and Figure 9.

**Figure 10:**
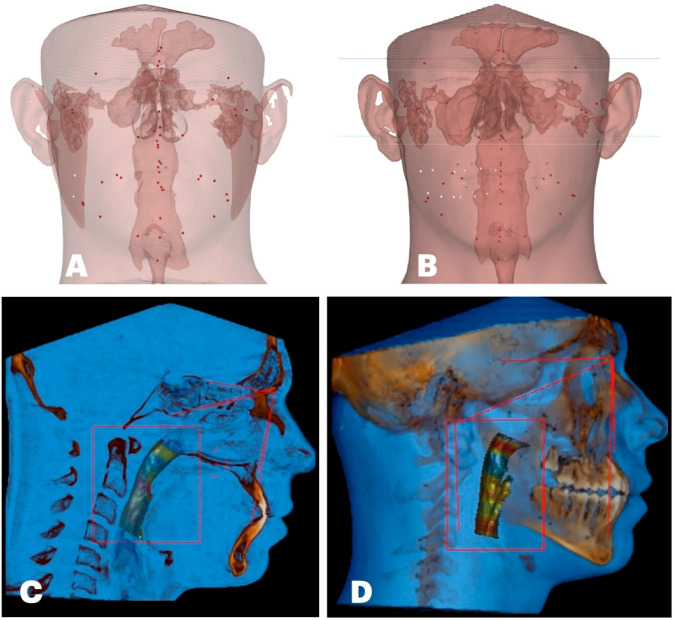
Size and orientation of the upper airway in the coronal plane: (**A**) before treatment; (**B**) after midfacial expansion. Size and orientation of the upper airway in the sagittal plane: (**C**) inclination of the upper airway with respective cervical lordotic changes; (**D**) post-expansion cervical curvature improvement with an increase in the total upper airway volume and its minimum cross-section and a reduction in the forward upper airway inclination. Upper airway mesh is marked with colors corresponding to various minimum cross-section areas, with red corresponding to the minimum cross-section area and blue corresponding to the maximum measured diameter.

Upper airway changes are rendered in Figure 10.

## 4. Discussion

Most authors have reached consensus regarding the favorable outcomes of MARPE [18,19], in combination with a facemask and/or alternating expansion and constriction in activating the appliance, for patients exhibiting features of Skeletal Class III malocclusion.

The majority of studies evaluating the effects of MARPE refer to cephalometric measurements derived from lateral and frontal cephalometric views. Thus, nasal cavity width, molar inclination, and intermolar width are the most widely used parameters in the frontal plane [6,20,21], while sagittal changes are evaluated when assessing the efficiency of MARPE in correcting Skeletal Class III discrepancies [18,19].

The three-dimensionally guided midpalatal piezocorticotomy technique has been described by the authors of a case series and a case report [5,14], emphasizing the importance of minimal invasiveness and a rigorous planning process to avoid multiple side effects, including, but not limited to, asymmetrical displacement of the nasal septum, asymmetrical maxillary expansion, and perimaxillary suture fractures. As reported by Kaya and coauthors [22], tooth–bone-borne MARPE appliances tend to deliver more stress to the teeth integrated into the framework, while bone-borne MARPE appliances deliver more stress to the surrounding bone. In their study, the levels of bone stress correlated with the degree of midpalatal separation, increasing stress concentration in the area of the pterygomaxillary junction and transverse suture. This potentially increases the risk of fractures in corresponding areas. Three-dimensionally guided midpalatal piezocorticotomy, on the other hand, is pre-planned through locating the ANS, PNS, and exact junction between the nasal septum and the maxillary crests. This allows the stress distribution in surrounding areas to be diminished.

Historically, MARPE treatment has been compared to Rapid Palatal Expansion (RPE) treatment in primary and mixed dentition. First described by Haas [23], midpalatal suture separation caused downward and forward movement of the maxilla. Davis and Kronman agreed in this regard [24], while de Silva reported no forward movement but explicit downward movement of the maxilla [25]. Conversely, Wertz and Dreskin [26] reported downward and backward rotation of the palatal plane.

To the best of our knowledge, no studies to date have evaluated the 3D changes in skeletal structure following MARPE or 3D-guided midpalatal piezocorticotomy-assisted MARPE treatment. The present case report focuses on the 3D analysis of changes following 3D-guided midpalatal piezocorticotomy-assisted MARPE, which had previously been shown to preserve the nasal septum’s position while removing the component of it restricting midpalatal disarticulation [5]. Standardized head orientation is essential in evaluating the effects of MARPE, with cervical curvature and postural changes being concomitant with midpalatal disarticulation. In our observations, cervical kyphotic changes were reversed due to an increase in nasopharyngeal airway volume. This has been reported in multiple studies, including long-term observational studies on MARPE [6,27].

The current study highlights rotational changes to the ANS–PNS plane in several spatial planes, including the sagittal plane, where anterior downward rotation took place, along with posterior upward rotation around the maxilla. This finding must be carefully interpreted in light of multiple-suture disarticulation following 3D-guided midpalatal piezocorticotomy-assisted MARPE. Previous studies reporting downward rotation of the palatal plane used SN-ANS-PNS angular measurement to evaluate changes [23,24,25,26]. One lateral cephalometric study conducted after SARPE reported that the pre-treatment palatal-plane angle was a predictor of downward rotation of the palatal plane post procedure. [28] We have observed disarticulation of the frontonasal and frontomaxillary sutures with larger magnitudes of midpalatal separation following 3D-guided midpalatal piezocorticotomy-assisted MARPE. This yields the assumption that nasal bone may undergo downward displacement, contributing to the changes in SN-associated angles. The current report also shows a decrease in anterior facial height.

The ANS-PNS plane rotated to the left in the coronal plane, with the center of rotation approaching the PNS. Sagittal rotation of the ANS–PNS plane has been reported previously by Cantarella and colleagues [29], while several studies have confirmed the downward and forward displacement of the zygomaticomaxillary complex following midfacial expansion [12,30]. To the best of the authors’ knowledge, the coronal-plane orientation of the ANS–PNS plane has not yet been described in the literature.

Maxillary cant, maxillary canine cant, and molar cant are evaluated in planning Maxillo-Mandibular Advancement (MMA) surgical procedures, including Le Fort I surgical procedures. In this study, maxillary canine cant was specifically evaluated to compare its vector to the ANS–PNS orientation and mandibular rotation. The total vector of the ANS–PNS rotation observed in this study was oriented upward and outward, increasing the size of the left nasal passage, left maxillary sinus, and left maxillary molar width and rotating all three planes upward (Table 4). Thus, measurements derived from the sagittal CBCT slices alone cannot accurately describe the combined changes in the maxillary and midfacial structures. In previous studies, maxillary-plane rotational changes were primarily derived from midfacial sagittal slices, while transverse changes were evaluated based on coronal slices at the level of the first maxillary molars and frontozygomatic or zygomaticomaxillary sutures. This case report provides preliminary evidence supporting the need to further evaluate 3D changes occurring as a consequence of MARPE, particularly 3D-guided midpalatal piezocorticotomy-assisted MARPE, and to interpret changes derived from superimposed lateral cephalometric images with greater care and accuracy.

A limitation of the current study is its reliance on limited data, comprising a single case report with no reported follow-up.

However, it is worth noting that the patients undergoing the procedure described herein will be followed for a subsequent 18–24 months as a part of ongoing treatment. A previous study performed by the same group of authors reported stable bone width increase in the area undergoing midpalatal separation over a 24-month follow-up period [5].

Further studies are necessary to confirm and corroborate the reported findings.

## 5. Conclusions

Three-dimensionally guided midpalatal piezocorticotomy-assisted MARPE has been shown to produce midfacial changes in all three spatial planes when evaluated via 3D cephalometric analysis. Comprehensive observational studies are necessary to analyze these changes and their effects for different skeletal classifications.

## 6. Patents

US and Canada Patent Pending: Piezocorticotomy guide for midpalatal skeletal expansion (Application # 18/919,416).

## Figures and Tables

**Table 1:** Lateral cephalometric measurements and their values before treatment (Roth–Jarabak analysis, modified).

Measurement	Value, Degrees
SNA	88.9
SNB	87.7
ANB	1.3
SN-ANS-PNS	0.9
FH-ANS-PNS	−9.1
SN-GoMe	27.8
ANS-PNS-GoMe	26.9
U1 to Palatal Plane	113.8

**Table 2:** Lateral cephalometric measurements and their values after expansion.

Measurement	Value, Degrees
SNA	88.4
SNB	85.6
ANB	2.8
SN-ANS-PNS	3.1
FH-ANS-PNS	−8.5
SN-GoMe	28.3
ANS-PNS-GoMe	25.2
U1 to Palatal Plane	114.2

**Table 3:** Three-dimensional cephalometric measurements used for analysis and their descriptions.

Measurement	Description
ANS to TVL	Distance from ANS to True Vertical Plane (TVL) Through Sn (Subnasal)
Mx Incisor R to TVL	Distance from Maxillary Right Central Incisor Tip to TVL (Sn)
Mx Incisor L to TVL	Distance from Maxillary Left Central Incisor Tip to TVL (Sn)
Upper-Right Canine to TVL	Distance from Maxillary Right Canine Tip to TVL (Sn)
Upper-Left Canine to TVL	Distance from Maxillary Left Canine Tip to TVL (Sn)
Upper-Right Molar to TVL	Distance from Maxillary Right First Molar Mesiofacial Cusp Tip to TVL (Sn)
Upper-Left Molar to TVL	Distance from Maxillary Left First Molar Mesiofacial Cusp Tip to TVL (Sn)
Pogonion to TVL	Distance from Pogonion to TVL (Sn)
B-Point to TVL	Distance from Point B to TVL (Sn)
PNS to TVL	Distance from PNS to TVL (Sn)
Mx33 Cant	Cant of the Maxillary Canine Plane (Positive—Right-Side Down; Negative—Left-Side Down)
Width Difference Mx Molar	Width Difference Between Right and Left Measurements of Maxillary Molar Width
Width Difference Zyg Arch	Width Difference Between Right and Left Measurements of Zygomatic Arch Width
Width Difference Lat Orb Rim	Width Difference Between Right and Left Measurements of Lateral Orbital Rim Width
Mx Molar R Width	Distance Between Distofacial Cusp of Maxillary Right First Molar and Midfacial Plane
Mx Molar L Width	Distance Between Distofacial Cusp of Maxillary Left First Molar and Midfacial Plane
Zyg Arch R Width	Distance Between Right Zygomatic Arch and Midfacial Plane
Zyg Arch L Width	Distance Between Left Zygomatic Arch and Midfacial Plane
Lat Orb Rim R Width	Distance Between Right Frontozygomatic Suture and Midfacial Plane
Lat Orb Rim L Width	Distance Between Left Frontozygomatic Suture and Midfacial Plane
Mx Incisor Height	Distance Between THP (Na) and Maxillary Central Incisor Tip
Max Canine R Height	Distance Between THP (Na) and Maxillary Right Canine Tip
Mx Canine L Height	Distance Between THP (Na) and Maxillary Left Canine Tip
Mx Molar R Height	Distance Between THP (Na) and Maxillary Right First Molar Mesiofacial Cusp Tip
Mx Molar L Height	Distance Between THP (Na) and Maxillary Left First Molar Mesiofacial Cusp Tip
Pogonion Height	Distance Between Pogonion and THP (Na)
PNS Height	Distance Between PNS and THP (Na)
Menton Height	Distance Between Menton and THP (Na)
RChkbone	Soft Tissue Distance from Right Cheekbone to TVP (Sn)
LChkbone	Soft Tissue Distance from Left Cheekbone to TVP (Sn)
Rnasalbase	Soft Tissue Distance from Right Nasal Base to TVP (Sn)
Lnasalabase	Soft Tissue Distance from Left Nasal Base to TVP (Sn)

**Table 4:** Three-dimensional cephalometric measurements with values before and after expansion and their changes.

Measurement	Before	After	Difference
ANS to TVL	−15.1 mm	−10.7 mm	−4.4 mm
Mx Incisor R to TVL	−16.5 mm	−16.0 mm	−0.5 mm
Mx Incisor L to TVL	−19.6 mm	−15.7 mm	−3.9 mm
Upper-Right Canine to TVL	−21.0 mm	−18.5 mm	−2.5 mm
Upper-Left Canine to TVL	−26.2 mm	−21.7 mm	−4.5 mm
Upper-Right Molar to TVL	−36.8 mm	−36.2 mm	−0.4 mm
Upper-Left Molar to TVL	−41.3 mm	−41.4 mm	0.1 mm
Pogonion to TVL	−14.7 mm	−16.8 mm	2.1 mm
B-Point to TVL	−14.7 mm	−17.0 mm	2.3 mm
PNS to TVL	70.6 mm	68.1 mm	−2.5 mm
Mx33 Cant	−3.2 degrees	1.1 degrees	−2.1 degrees
Width Difference, Mx Molar	3.2 mm	−3.7 mm	−0.5 mm
Width Difference, Zyg Arch	3.5 mm	−2.6 mm	0.9 mm
Width Difference, Lat Orb Rim	−1.4 mm	−1.7 mm	0.3 mm
Mx Molar R Width	30.8 mm	32.4 mm	1.6 mm
Mx Molar L Width	27.7 mm	36.1 mm	8.4 mm
Zyg Arch R Width	67.6 mm	67.7 mm	0.1 mm
Zyg Arch L Width	67.5 mm	68.3 mm	0.8 mm
Lat Orb Rim R Width	45.6 mm	52.0 mm	6.4 mm
Lat Orb Rim L Width	46.9 mm	53.6 mm	6.7 mm
Mx Incisor Height	71.0 mm	74.2 mm	3.2 mm
Max Canine R Height	70.9 mm	73.0 mm	2.1 mm
Mx Canine L Height	74.1 mm	72.0 mm	−1.9 mm
Mx Molar R Height	72.7 mm	73.9 mm	1.2 mm
Mx Molar L Height	73.8 mm	73.1 mm	−0.7 mm
Pogonion Height	115.5 mm	117.6 mm	2.1 mm
PNS Height	55.5 mm	55.3 mm	−0.2 mm
Menton Height	121.8 mm	124.2 mm	2.4 mm
RChkbone	41.1 mm	35.4 mm	−5.7 mm
LChkbone	39.0 mm	37.3 mm	−1.7 mm
Rnasalbase	0	0	0
Lnasalabase	0	0	0

**Table 5:** Upper airway measurements before and after expansion.

Measurement	Before	After
Upper Airway Total Volume, cc	19.5	21.7
Upper Airway Min Cross-Section, mm^2^	253	307

## Data Availability

The original contributions presented in this study are included in the article. Further inquiries can be directed to the corresponding author.

## Abbreviations

The following abbreviations are used in this manuscript:

MARPE	Mini-Screw-Assisted Rapid Palatal Expansion
ANS	Anterior Nasal Spine
PNS	Posterior Nasal Spine
CBCT	Cone-Beam Computed Tomography
MSE	Maxillary Skeletal Expander
